# Inhibition of the Nicotinic Acetylcholine Receptors by Cobra Venom α-Neurotoxins: Is There a Perspective in Lung Cancer Treatment?

**DOI:** 10.1371/journal.pone.0020695

**Published:** 2011-06-13

**Authors:** Angela Alama, Cristina Bruzzo, Zita Cavalieri, Alessandra Forlani, Yuri Utkin, Ida Casciano, Massimo Romani

**Affiliations:** 1 Lung Cancer Unit, Istituto Nazionale per la Ricerca sul Cancro, IST, Genova, Italy; 2 Laboratory of Tumor Genetics and Epigenetics, Istituto Nazionale per la Ricerca sul Cancro, IST, Genova, Italy; 3 Shemyakin-Ovchinnikov Institute of Bioorganic Chemistry, Moscow, Russia; Queen Elizabeth Hospital, Hong Kong

## Abstract

Nicotine exerts its oncogenic effects through the binding to nicotinic acetylcholine receptors (nAChRs) and the activation of downstream pathways that block apoptosis and promote neo-angiogenesis. The nAChRs of the α7 subtype are present on a wide variety of cancer cells and their inhibition by cobra venom neurotoxins has been proposed in several articles and reviews as a potential innovative lung cancer therapy. However, since part of the published results was recently retracted, we believe that the antitumoral activity of cobra venom neurotoxins needs to be independently re-evaluated.

We determined the activity of α-neurotoxins from *Naja atra* (short-chain neurotoxin, α-cobrotoxin) and *Naja kaouthia* (long-chain neurotoxin, α-cobratoxin) *in vitro* by cytotoxicity measurements in 5 lung cancer cell lines, by colony formation assay with α7nAChRs expressing and non-expressing cell lines and *in vivo* by assessing tumor growth in an orthotopic Non-Obese Diabetic/Severe Combined Immunodeficient (*NOD/SCID*) mouse model system utilizing different treatment schedules and dosages.

No statistically significant reduction in tumor growth was observed in the treatment arms in comparison to the control for both toxins. Paradoxically α-cobrotoxin from *Naja atra* showed the tendency to enhance tumor growth although, even in this case, the statistical significance was not reached.

In conclusion our results show that, in contrast with other reports, the nAChR inhibitors α-cobratoxin from *N. kaouthia* and α-cobrotoxin from *N. atra* neither suppressed tumor growth nor prolonged the survival of the treated animals.

## Introduction

The experimental evidences suggesting that stimulatory or inhibitory neurotransmission is involved in cancer development, progression and in the response to therapy have steadily accumulated [1]. Indeed, tobacco components regulate cellular functions related to cell transformation and are involved with smoking addiction and lung cancer predisposition and development by directly interacting with neuronal and non-neuronal nicotinic acetylcholine receptors (nAChRs) [2], [3], [4], [5], [6], [7], [8], [9], [10], [11]. Nicotine itself has limited lung cancer initiating capabilities but can sustain tumor growth and promote metastatic spread through its antiapoptotic and neoangiogenic properties [2], [7], [12].

The expression of nAChRs in non-neural cells of the lung, and particularly in the airway epithelium, reflects the multiple essential functions exerted by the cholinergic system in normal lung development and function [13], [14]. In this respect, it has been proposed that the role of nAChRs in lung cancer might be similar to that of estrogen receptors in breast cancer since in both cases the inappropriate stimulation of the receptors contributes to cancer development [15]. In view of the high level of expression of certain subtypes of nAChRs in lung cancer cells compared to the surrounding unaffected tissue [16], [17] and of supporting experimental evidences [18], [19], [20], [21], it was hypothesized that antagonists of nAChRs, and in particular cobra α-neurotoxins, could be exploited as potential therapeutic agents [22], [23], [24]. However, the limited knowledge of the functionality and of the long-term effects of the stimulation of these receptors in cancer cells [2], [25], [26] and, most importantly, the recent retraction of a report supporting the antitumoral effects of cobra α-neurotoxins *in vitro* and *in vivo* [27] renders questionable the targeting of nAChRs with these toxins for lung cancer treatment.

Cobra venom is constituted by many polypeptides with multiple toxic activities. Among these, the three-fingered toxins (TFTs) are the main components and are represented by α-neurotoxins and cytotoxins. The α-neurotoxins bind to nAChR with different specificity and affinity: short-chain toxins (60–62 amino acid residues, 4 disulfide bridges) block muscle-type nAChRs whereas long-chain toxins (66–75 amino acid residues, 5 disulfide bonds, the fifth bond being present in the central polypeptide loop) in addition to muscle-type nAChRs block also the neuronal receptors [28], [29]. As an example of short-chain toxins, α-neurotoxin called α-cobr*o*toxin from *Naja atra* cobra venom can be mentioned. The principal α-neurotoxin from *Naja kaouthia* cobra venom called α-cobr*a*toxin is an example of long-chain toxins. The short-chain toxins are structurally related to the cytotoxins that non-selectively kill the cells [30].

While reviewing the literature on the anti-tumor effects of α-cobratoxin [18], [19], [20], [21], [22], [27] we realized that the various reports presented major differences on the dosage of the toxin utilized for the *in vivo* experiments, on the number of injected cells and on mice survival. Also the presence of the α7 nAChR on the cell line utilized for the in vivo studies was uncertain since conflicting results are present in the literature [22], [31]. Furthermore, since part of the data were retracted with no specific motivation [27], we felt necessary to re-evaluate the anti-cancer properties of cobra neurotoxins *in vitro* and *in vivo* in a clinically relevant animal model of lung cancer [18] to clarify if these toxins could be considered the prototype of a novel class of natural products with antitumor properties as proposed [15], [22], [24].

## Results and Discussion

### Expression of α7 nAChR in A549 and A549-luc cells

**Figure 1:**
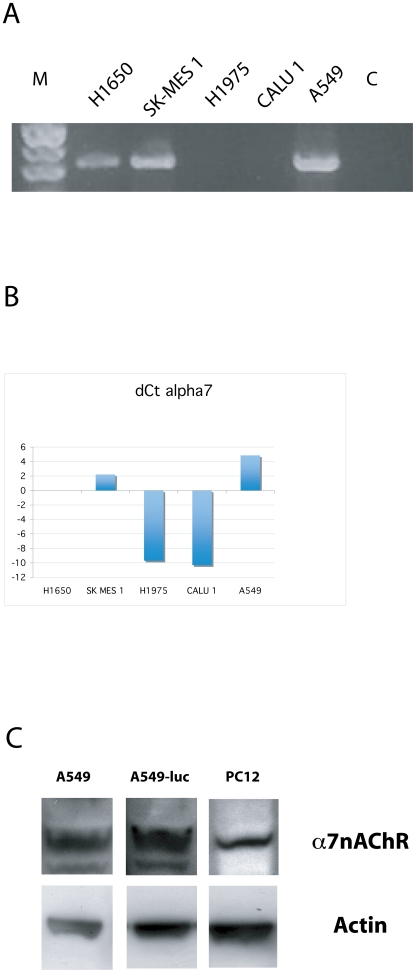
Presence of the α7-nACh receptor on NSCLC cell lines. A) Semiquantitative Alpha7 RT-PCR analysis on Human NSCLC adenocarcinoma e squamous cell carcinoma cell lines. GAPDH expression was utilized as internal control for mRNA integrity and cDNA quantification. NCI-H1975 and Calu1 are negative. C: No template control. B) qPCR alpha 7 expression in the same cell lines. On y-axis natural logaritm (ln) of fold change. NCI-H1650 was used as calibrator, GAPDH as reference gene. One ugr of Total RNA was retrotrascibed and the same amount of cDNA per sample (2 ul) was used (Ct GAPDH comprised between 15.65 and 17.65). NCI-H1975 and Calu1 resulted negative or with extremely low expression due to Ct>40. C) Western blot analysis for alpha 7. PC12 was loaded as positive control. Actin expression was utilized as internal control.

The interaction between α-cobratoxin and the α7 nicotinic receptor [32] was one of the experimental evidences behind the rationale of utilizing cobra venom toxins as anticancer agents [22]. Although this receptor is expressed on a wide spectrum of tissues and cell lines, a recent survey of the literature [22], [31] reported conflicting data on the expression of this receptor in A549, the cell line utilized for the *in vivo* and most of the *in vitro* anticancer assays on α-cobratoxin. Therefore, as an initial step to verify the activity of α-cobratoxin in NSCLC, we performed a semiquantitative RT-PCR and qPCR survey to demonstrate the expression of α**7** nAChR in 5 lung cancer cell lines. Three of the cell lines utilized in the present study (A549, H1650 and SK-MES 1) were the same of the original set of experiments [18], [19], [20], [27]. As shown in Figure 1, Panel A, the α7 nicotinic receptor was readily detectable in A549, H1650 and SK-MES 1 but not in H1975 and CALU 1. The qPCR analysis confirmed the presence of different amount of the α7 nAChR mRNA in A549, H1650 and SK-MES 1 (Figure 1, Panel B). In agreement with the RT-PCR results, in H1975 and CALU 1 the α7 nAChR transcript, using the same amount of cDNA used for all the cell lines, appeared after cycle 40, a result that could be attributed either to an extremely low level of expression or to background noise

The expression of α7 nAChR at the protein level was confirmed by western blot analysis in A549 and A549-luc cells (Figure 1, Panel C).

It has been reported that stimulation of α7 nAChR in human lung cancer cells results in the up regulation of this receptor [33]. We have tested the influence of the α-neurotoxins from *N. atra* and *N.kaouthia* on the level of α7 nAChR focusing on A549 and A549-luc.

The qPCR analysis showed that the treatment with either α-cobrotoxin or α-cobratoxin at the concentration of 0.003 µM, the reported IC_50_ for α-cobratoxin in A549 [20], together with concentrations three times lower (0.001 µM) and three times higher (0.009 µM), did not substantially change the level of expression of the receptor in these cell lines (less than two-fold change, Figure S1).

### In vitro effects of short- and long-chain α-neurotoxins on NSCLC cell lines

Early *in vitro* experiments suggested that the effect of α-cobratoxin is dose dependent and that the high selectivity and specificity of this molecule depends from the density of α7 nAChR [20], [21]. In this respect non-tumoral pulmonary cells as well as other primary unaffected cells expressing low levels of α7 receptors were remarkably resistant to α-cobratoxin treatment [20].

To evaluate the cytotoxic activity of α-cobratoxin, that efficiently binds to the α7 nAChR (see Materials and Methods) and the specificity and selectivity of its action, we performed MTT assays with the short- and long-chain α-neurotoxins on presumably sensitive α7 nAChR-positive and presumably resistant α7 nAChR–negative cell lines. Dose-response curves were drawn to assess the drug concentration reducing survival. The initial cytotoxicity experiments were carried out utilizing toxins concentrations that in other publications were reported to be effective in α7 nAChR-positive NSCLC cell lines but not toxic in normal cells (IC50: 0.003 µM for A549, 0.04 µM for SK-MES 1 and 1 µM for H1650) [18], [20], [21], [22]. However, at these concentrations we could not detect appreciable cytotoxic activity and the IC50 could not be reached with any of the two toxins (data not shown).

**Figure 2:**
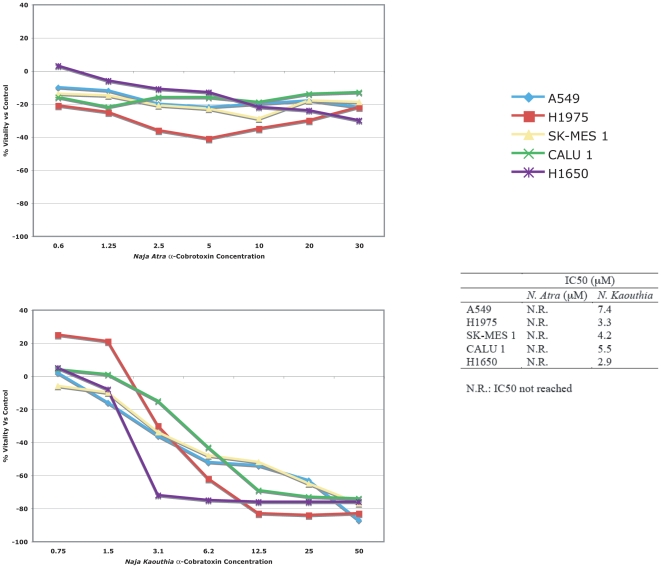
Cytotoxicity assay. The NSCLC cell lines A549, NCI-H1975, H1650, CALU 1 and SK-MES 1 were incubated for 72 hours with **α**-cobrotoxin (0.6–30 µM), upper panel, or **α**-cobratoxin (0.75–50 µM), lower panel, and the toxicity was measured with the colorimetric MTT test. The IC50 was reached only with **α**-cobratoxin.

At higher concentrations (up to 30 µM) the α-cobrotoxin showed a limited non dose-dependent toxicity that remained essentially constant in a wide range of concentrations in all 5 cell lines (Figure 2, Panel A).

At high concentrations the α-cobratoxin showed a clear dose-dependent toxicity (Figure 2, Panel B). However the IC50 concentration observed in our study for A549 and SK-MES 1 was 2466 and 105 fold higher than that reported in an earlier publication [20]. The difference was much lower for H1650 (2.9 fold) a result compatible with the utilization of a different toxin preparation.

The limited toxic effect of the short chain α-cobrotoxin could be explained by its inability to bind to the α7 receptor. Surprisingly however, the α-cobratoxin was effective also on α7 nAChR-negative cells suggesting that the toxic activity is not mediated by the binding of the toxin to the α7 receptor.

**Figure 3:**
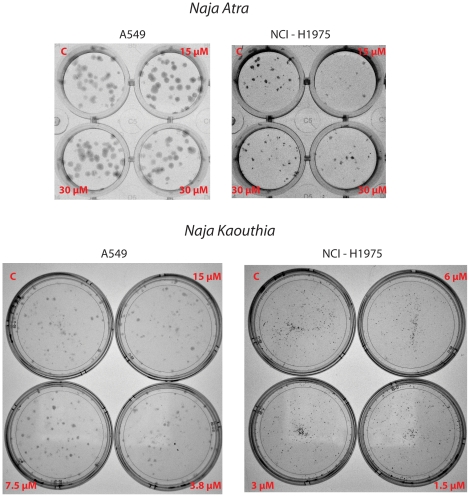
Clonality assay. The cell lines A549 (α7 AChR +) and NCI-H1975 (α7 AChR -) were plated in 35 mm Petri dishes or in 24 wells plates at densities of 150 (A549) and 300 (NCI-H1975) cells/dish and exposed to either long-chain α-neurotoxin from *Naja Kaouthia*, at concentrations of 15 µM, 7.5 µM, 3.8 µM (A549) and 6 µM, 3 µM, 1.5 µM (NCI-H1975), or short-chain from *Naja Atra*, at concentrations of 30 µM and 15 µM (both cell lines). Treated cells were then incubated for 7–10 days, until visible colonies formed.

To confirm and extend this observation we have conducted a colony formation assay with the α7 nAChR+A549 and with the α7 nAChR−NCI-H1975 cell lines . Since the IC50 was not reached with α-cobrotoxin, we utilized the two highest concentrations tested by MTT (15 and 30 µM). For α-cobratoxin we utilized the concentrations corresponding to the IC50 for A549 and NCI-H1975 (7. 5 and 3 µM, respectively) and concentrations corresponding to half and twice the IC50. As shown in Figure 3, the clonogenic activity of the two cell lines was not affected by the treatment and by the presence of the α7 nACh receptor.

The activation of the apoptotic cascade was considered the key effect of the binding of the α-cobratoxin to the α7 nAChR [18], [20], [21], [22]. In view of the major differences between our results and those previously published regarding the dosage at which the IC50 could be obtained and the absence of selective action of α-cobratoxin on α7 nAChR-positive cells, we tried to understand if activation of apoptosis indeed occurs upon treatment with α-cobratoxin by Annexin V-PI flow cytometry staining. It was reported that the maximum induction of apoptosis in A549 cells could be obtained by treatment with 1 µM toxin for 24 hours [18]. We have repeated the same experiment utilizing the same methodology but with toxin concentrations (1, 10 and 50 µM) that induced growth inibition ranging from 5 to 87% in MTT assays.

**Figure 4:**
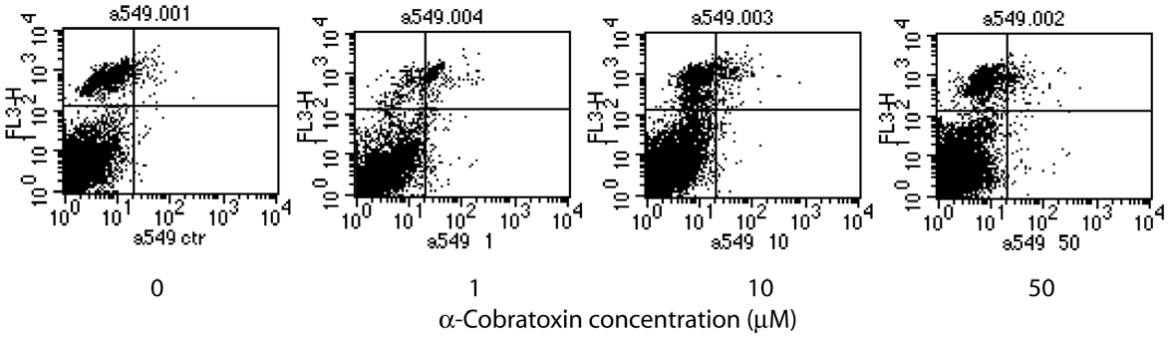
Evaluation of the Apoptosis elicited by α-cobratoxin in A549 cells. Cells were incubated for 24 hours with 1, 10 and 50 µM **α**-cobratoxin and apoptosis was evaluated by Annexin V-PI staining and FACS separation. Apoptosis in untreated cells was 0.63% and in treated cells was in the range 0.98–2.32%.

As shown in Figure 4, even at the highest concentration very few cells (1–3%) presented evidences of apoptosis with necrotic cells being prevalent.

Overall these results suggest that the cell death observed in vitro is likely the result of necrosis rather than the activation of the apoptotic pathway following the specific binding of α-cobratoxin to its ligand.

### *In vivo* toxicity of α-neurotoxins

The acute toxicity of increasing doses of toxins after i.v. administration (as in M&M) was assessed in CD1 mice on the basis of clinical and behavioral signs. Acute symptoms developed within 15 minutes after injection and were mostly characterized by general discomfort and respiratory distress that restored to the normal conditions within 60–180 minutes. Based on the number of dead CD1 mice, following administration of either α-cobrotoxin or α-cobratoxin and over an observation period of 14 days, the LD values were determined and the MTD identified as 0.1 mg/kg for α-cobrotoxin and 0.2 mg/kg for α-cobratoxin as shown in Table 1.

**Table 1:** In vivo toxicity of α-cobrotoxin and α-cobratoxin.

CbTx (mg/kg) i.v.	Dead/Overall LD	Dead/Overall LD
	α-cobrotoxin		α-cobratoxin	
5	1/1	100		
1	1/1	100		
0.5	1/1	100	3/3	100
0.4			2/3	60
0.35			2/4	50
0.30			1/3	30
0.25	2/2	100		
0.20	4/4	100	0/2	MTD
0.16	4/4	100		
0.135	4/5	80		
0.130	3/4	75		
0.128	2/4	50		
0.125	1/6	16	0/2	-
0.100	0/2	MTD	0/2	-

Importantly, the LD50 for α-cobrotoxin determined in our study (0.128 mg/kg) was in very good agreement with literature data (0.1 mg/kg: http://www.uniprot.org/uniprot/P60770). The observed LD50 for α-cobratoxin was 0.35 mg/kg, a value in the same order of magnitude of the literature data (0.1 mg/kg; http://www.uniprot.org/uniprot/P01391).

The reports on the in vivo toxicity of α-cobratoxin are confusing and in the recent literature we have observed that the LD50 concentration reported in three different publications from the same group differs of 1000 fold (0.15 mg/kg or 0.15 µg/kg) [18], [21], [22]. The results obtained in the present study are essentially in agreement with those of two of the previous reports [18], [21] and, importantly, demonstrate the biological activity *in vivo* of our toxin preparations.

Half of the MTD for each toxin (0.05 and 0.1 mg/kg, respectively) were then used in one of the two treatment protocols designed to assess the antitumor activity in vivo of these toxins.

### Antitumor activity of α-neurotoxins

To assess the antitumor activity of both toxins, *NOD/SCID* mice were orthotopically transplanted with 0.5×10^6^ human A549-luc cells/in a volume of 10 µl PBS into the right lung. As surrogate marker of tumor growth we measured the light emission of the luciferase-tagged A549 cells; this system enabled us to longitudinally follow each animal and to monitor the effect of the treatment.

**Figure 5:**
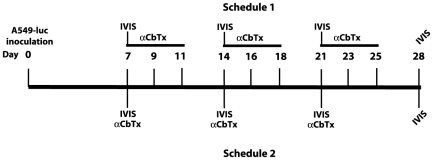
Treatment schedules for *in vivo* studies. Mice were injected with 0.5×10^6^ human A549-luc cells. At day 7, after the IVIS determination, the animals were randomized and subjected to treatment. For schedule 1 the mice were treated with 1/1000 of the LD10 as indicated in [20] three times/week for three weeks. For schedule 2 the mice were treated once a week with a dosage corresponding to ½ MTD (determined in the present study).

Following the assessment of tumor implants, by IVIS detection, the mice were randomly assigned to one of the study groups and treatment started 7 days after orthotopic transplantation according to the two different protocols outlined in Figure 5.

Protocol 1: 8 animals were assigned to receive i.v. 0.12 µg/kg of toxin (1/1000 of the LD_10_ indicated in [20]), three times a week for three weeks according to a previously reported schedule [20] and 8 animals received vehicle alone.

Protocol 2: 8 animals were treated i.v. once a week for three weeks with of 0.05 mg/kg of α-cobrotoxin or 0.1 mg/kg of α-cobratoxin (corresponding to half-MTD). For α-cobratoxin the half-MTD corresponds to 12.8 µM, a concentration that *in vitro* should kill between 50 and 63% of the A549 cells. Eight control animals received vehicle alone.

In another publication [18] where the time-schedule of protocol 1 was utilized, the reported **α**-cobratoxin dosage was 0.12 mg/kg. On the basis of our *in vivo* toxicity determination we considered this dosage too high to be administered for three consecutive days to mice with compromised respiratory function.

After an initial assessment of tumor growth at day 7, the antitumoral activity induced by toxins was evaluated in treated and untreated mice, at days 14, 21 and 28 post-administration in both protocols. We have observed that at the initial stages of growth, the bioluminiscence of the animals closely represented the extent of tumor growth. Conversely, at later stages, tumor necrosis and reduced perfusion, likely diminished the light emission in mice even when the tumor had completely invaded the thoracic cavity and, in most cases, had extended outside the thorax.

The pre-treatment IVIS evaluation of the 56 mice included in the study at day 7 showed successful tumor growth in all animals in spite of a 300-fold individual variability (average photon emission: 5.5×10^7^/mouse, range 6.68×10^5^−2.06×10^8^). Therefore, for data analysis, we considered the fold-change of emission between treated and non-treated animals at each treatment time point (14, 21 and 28 days) rather than the absolute photon emission values [18].

**Figure 6:**
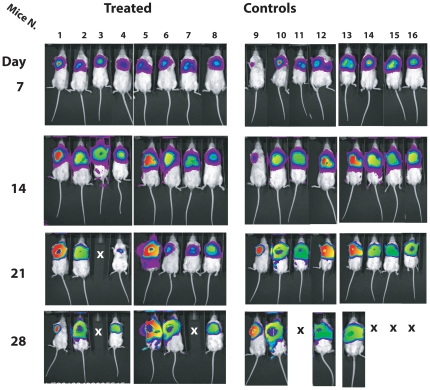
Raw IVIS determination of tumor growth in mice treated with α-cobrotoxin according to schedule 1. X denotes the dead animals.

**Figure 7:**
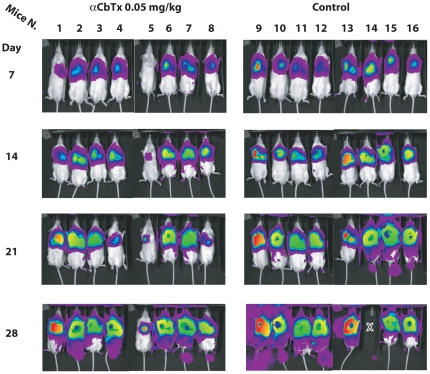
Raw IVIS determination of tumor growth in mice treated with α*-*cobrotoxin according to schedule 2. X denotes the dead animals.

In Figure 6, we report the raw IVIS determination in each mice treated with α-cobrotoxin according to schedule 1 and in the corresponding control group. As shown, this treatment does not seem to have an appreciable effect on tumor growth in this animal model system. The only apparent striking difference was the number of dead animals at the end of the observational period (4 in the control group and 2 in the treatment group). However, it must be noticed that at this time all animals had to be sacrificed for ethical reasons and that autopsy revealed that the tumor had extended outside the thoracic cavity in all treated and untreated mice. As shown in Figure 7 also at the higher dosages utilized in the schedule 2 treatment plan, the effect of α-cobrotoxin as antitumor agent was negligible, if any. As for the schedule 1 treatment plan, also these animals were sacrificed at day 28 because of tumor growth-related symptoms. Indeed, at autopsy all animals presented a completely invaded thoracic cavity with tumors extending outside the chest irrespective of treatment scheme.

**Figure 8:**
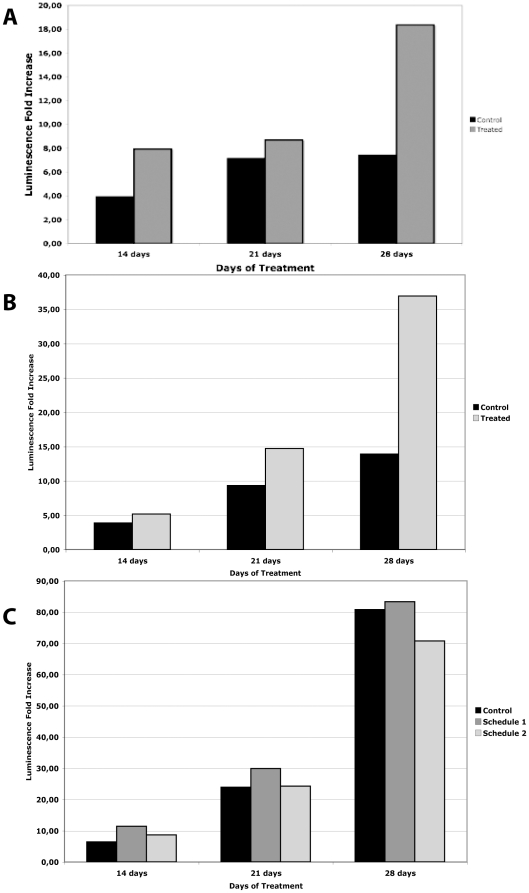
Fold-Change of bioluminescence emission in tumor-bearing mice. Panel A: mice treated with **α**-cobrotoxin according to schedule 1. Panel B: mice treated with **α**-cobrotoxin according to schedule 2. Panel C: mice treated with **α**-cobratoxin according to schedules 1 and 2.

**Figure 9:**
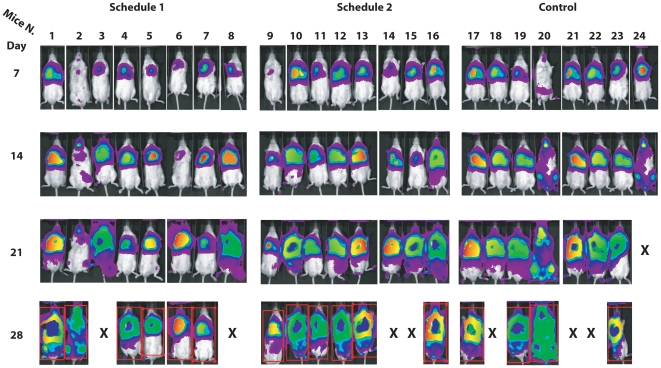
Raw IVIS determination of tumor growth in mice treated with α-cobratoxin according to schedules 1 and 2. X denotes the dead animals.

In Figure 8 is reported the averaged fold-change of emission in the treatment and control arms for schedule 1 (Panel A) and schedule 2 (Panel B). Although the difference, evaluated by the Mann-Withney test, did not reach statistical significance (except for schedule 1, day 14, p = 0.04), we observed a striking higher emission in the toxin treatment group with respect to the control arm. This effect was dramatically evident in the schedule 2 at day 28, although, the difference in the photon emission did not reach statistical significance, likely because of the limited number of animals,

The same treatment schedules were also utilized with the α-cobratoxin. The results of this set of experiments showed that this toxin lacks evident antitumoral activity as well. In Figure 9, we report the raw IVIS determination for each mice treated with α-cobratoxin according to schedules 1 and 2 while in Figure 8, Panel C, we show the average fold-change of emission in the control and treated mice. As can be seen, the treatment does not appreciably influence tumor growth as photon emission in treated mice was comparable to that of the controls at each time-point. The number of animals that had to be sacrificed for humanitarian reasons before the end of the observational period was higher in control as compared to treated mice. However, it must be pointed out that also in this case all animals, at day 28, presented massive tumor growth that extended outside the thoracic cavity irrespectively of the treatment plan.

The *in vivo* experiments were conducted only with the A549-luc cell line since the utilization of non-luc tagged cells would have required a large number of mice to assess the antitumor activity of the toxins. In view of the results obtained *in vitro* on 5 cell lines and *in vivo* with A549-luc we considered unethical to precede with additional animal studies

The molecular basis of lung cancer have been extensively studied and the interaction between nicotine and nAChRs has been recognized as one of the key events leading to the development of this tumor [2], [4], [5], [6], [7], [8], [9], [10], [11], [16], [26]. It is thus not surprising that nAChRs were considered as good candidate targets for innovative “biologic” therapies [15], [22], [23], [24]. In this respect, the existence of potent toxins inhibiting nACh receptors might open the possibility of adapting the Paul Ehrlich's “magic bullet” concept to lung cancer therapy [34].

The cobra snake venom is a source of different toxins possessing cytolytic and nAChR inhibiting activities [32], [35]. Because of this latter activity, the α-cobratoxin from *N. kaouthia* has been proposed as an innovative natural therapeutic agent for lung cancer [18], [19], [20], [22], [24] capable of dramatically inhibit lung tumor cells growth and to significantly improve the survival of lung tumor-bearing mice. We have re-evaluated the antitumoral activities of two different cobra neurotoxins *in vitro* and *in vivo* utilizing two schedules of administration and the same experimental conditions of previous reports. The results of our experiments clearly show that these two biologically active toxins have essentially no effects on tumor cell growth in *in vitro* assays at the concentration reported in other publications. A significant tumor cell growth inhibition was obtained at α-cobratoxin concentration too high to be utilized *in vivo*. Importantly α-neurotoxins, at concentrations usable *in vivo*, did not inhibit tumor growth nor were capable of significantly prolonging the survival in mice bearing an orthotopically-grafted NSCLC. Indeed, the *in vivo* experiments had to be interrupted at day 28, or earlier, in treated and untreated experimental animals for ethical reasons since in all cases the grafted tumor had extended outside the thorax. These results are in striking contrast with those of other studies that reported an increased lifespan of 93% in α-cobratoxin treated versus untreated mice [20] and suggest that cobra α-neurotoxins have no potential therapeutic effect in lung cancer.

It remains to be explained why in one published report from the same group all animals had to be killed for humanitarian reasons at day 29 [18] while in another study the animals, similarly treated, could be followed up to day 170 [20], (reviewed in [22]).

Surprisingly we have observed that the *in vivo* tumor growth in the mice treated with **α**-cobrotoxin was higher than that of the control groups. This unexpected finding might suggest that although this toxin does not bind to the α7 receptor, the reported predominant subtype present on A549 cells, it might activate the nicotine-dependent tumorigenic pathway through its binding to different nAChR (most probably to muscle-type receptor). Thus, this toxin may be an excellent tool for finely dissecting the biological pathways where the nAChRs are involved.

## Materials and Methods

### Ethics statement

All the details about: permissions, regulations and animal welfare are reported in the “Animals” sub-section of this Methods section

### Tumour cell lines and α-neurotoxin preparations

The rat pheochromocytoma cell line PC12, utilized as positive control for α7 nAChR expression, the human NSCLC adenocarcinoma cell lines H1650 and H1975 were obtained from the ATCC; the NSCLC adenocarcinoma A549 and the squamous carcinoma cell lines SK-MES 1 and CALU 1, were obtained from our Institutional Cell Repository (ICLC, www.iclc.it). The cell line A549-luc, modified to stably express luciferase, and utilized for *in vivo* studies was kindly provided by Dr. J.W. Shay (H. Simmons Comprehensive Cancer Center, University of Texas, Dallas). The A549, A549-luc, SK-MES 1 and H1650 utilized in the present study had the same origin of those utilized in other published works [18], [19], [20], [27]. Cells were grown in RPMI 1640 with 10% bovine serum. (Invitrogen, San Giuliano Milanese, Italy).

The short-chain α-cobrotoxin from *N. atra* (MW 6949 kDa) was obtained from Sigma (Milano, Italy). The LD50 in mice of the batch utilized in this work, determined by the Company, was 0.09 mg/kg.

The long-chain α-cobratoxin (MW 7821 kDa) was purified from *N. kaouthia* venom as described [36]. The procedure involves: gel-filtration, high performance ion-exchange and reversed-phase chromatography and is utilized to produce large quantities of the toxin for receptor studies [37]. The α-cobratoxin prepared by this method is fully active and inhibited acetylcholine-induced currents in *Xenopus* oocytes expressing human α7 nicotinic acetylcholine receptor with IC_50_ of 4.1 nM [38]. The biologic activity of the batch of toxin utilized in this study was tested for ability to inhibit radioactive α-bungarotoxin binding to human α7 nicotinic acetylcholine receptor heterologously expressed in GH4C1 cells. At 20 nM α-cobratoxin inhibited α-bungarotoxin binding by more than 50%.

The lyophilized toxins were dissolved at the stock concentration of 10^−3^ M in Phosphate-Buffered Saline (PBS) and kept at −20°C.

### Western blot analysis

Cell suspensions were obtained after trypsinization of A549 or A549-luc and PC12 cultures (used as positive control). Cells were dissolved in lysis buffer and processed as previously described [39]. The protein concentration of cell lysates was determined by the Bio-Rad Protein Assay (Bio-Rad Laboratories, Segrate, Italy) according to the manufacturer's instructions.

Fifty µg of total proteins were separated on NuPAGE 4–12% bis-Tris gel (Invitrogen, San Giuliano Milanese, Italy) and then transferred onto a nitrocellulose membrane by iBlot™ Gel Tranfer Stacks (Invitrogen).

Blots were probed with the anti α7 AChR polyclonal antibody (Santa Cruz, Heidelberg, Germany) and subsequently with anti-ßActin monoclonal antibody (Sigma, Milano, Italy) to ascertain that an equal amount of protein was loaded in each lane.

Immunodetection was performed using the enhanced chemiluminescence (ECL) kit (GE Healthcare, Milano, Italy) following the supplier's recommended procedures.

### RT and qPCR analysis

Total RNA from A549-luc (untreated and treated with 1, 3 and 9 nM of α-cobratoxin or α-cobrotoxin for 72 h), A549 (untreated and treated with 1, 3 and 9 nM of α-cobratoxin), H1650, H1975 , SK-MES 1, CALU 1 and PC12 cell lines was isolated using the RNAeasy Mini Kit (Qiagen) following the manufacturer instructions. RNA was treated with RNAse-free DNAse during on-column purification. RNA integrity was assessed by gel electrophoresis: ratio of 28S to 18S was approximately 2∶1. RNA was quantified by spectrophotometry. The ratio of the readings at 260 nm and 280 nm was comprised between 1.9 and 2.1.

One µg of total RNA was used to prepare cDNA with the SuperScript™ II RNase H- Reverse Trascriptase (Invitrogen) according to the manufacturer instructions.

To determine the presence of α7 nAChR on cell lines, cDNA were used in a semiquantitative reaction as elsewhere described [40], [41], [42].

PCR conditions were: 95°C for 10 min, 45 cycles 95°C for 15 sec, 55°C for 15 sec and 72°C for 30 sec).

qPCR reactions were performed using Maxima Sybr Green qPCR Master Mix (Fermentas GMBH, St. Leon-Rot , Germany), 0.3 µM of each primer and nuclease-free water in a total volume of 25 µl. Relative expression was calculated using as calibrator A549-luc with ß2 Microglobulin as the reference gene. The reactions were performed using the Mastercycler ep RealPlex instrument and its analytical software (Eppendorf, Milano, Italy).

PCR conditions were: 95°C for 10 min, followed by 45 cycles as follow: 95°C for 15 sec, 50°C for 30 sec and 72°C for 30 sec. Specificity of the reaction was controlled by melting curve analysis ramping from 60°C to 95°C in 20 minutes.

Two independent quantitative PCR reactions were performed for each sample.

The sequences of the primers for RT and qPCR were: forward 5′-GCCAATGACTCGCAACCACTC-3; reverse 5′-CCAGCGTACATCGATGTAGCA-3′. These primers encompass bases 236–571 of the human α7 nAChR, Genbank accession number X70297 [33]. . The same primers are used also on PC12 positive control cell lines: human and rat target sequences have 84% overall homology and complete identity in the crucial 3′-terminal sequence.

### Cytotoxicity, colony formation assay and apoptosis detection *in vitro*

The colorimetric MTT (3-(4,5-dimethylthiazol-2-yl)-2,5-diphenyltetrazolium bromide) test was used to determine the sensitivity of the A549, SK-MES 1 (both 1.5×10^3^ cells) H1650, H1975 , and CALU 1 (3×10^3^ cells) cell lines towards increasing concentrations of toxins (from 0.6 to 50.0 µM) or CDDP (from 1.25 to 20.0 µM) (31). The IC values, defined as the concentrations inhibiting cell growth relative to control, were determined from the analysis of dose response-curves obtained after 72 h of exposure. Every concentration was tested in quadruplicate wells in each experiment and at least three independent experiments were performed.

The cell lines A549 (α7 AChR +) and NCI-H1975 (α7 AChR -), were grown in RPMI 1640 supplemented with 10% FBS. Cells were trypsinized and plated in 24-well plates or 35 mm Petri at densities of 50 and 100 or 150 and 300 cells/dish for A549 and NCI-H1975, respectively. Cells were allowed to adhere overnight and then exposed to either long- chain α-neurotoxin from *Naja Kaouthia*, at concentrations of 15 µM, 7.5 µM, 3.8 µM (A549) and 6 µM, 3 µM, 1.5 µM (NCI-H1975), or short-chain α-neurotoxin from *Naja Atra*, at concentrations of 30 µM and 15 µM (both cell lines).

Treated cells were then incubated for 7–10 days, until visible colonies formed. The dishes were then washed in phosphate-buffered saline, and colonies stained with 1% methylene blue in methanol for 15 min. Plates were then washed in tap water, and colonies containing greater than 50 cells were counted on an inverted microscope. Survival was compared to the plating efficiency of untreated controls.

Apoptosis was determined by annexin V–FITC and propidium iodide (PI) double staining (Bender MedSystem,Vienna, Austria), by fluorescence activated cell-sorting (FACS) (BD Biosciences, Milan, Italy) analysis [43]. A5409 cells (2.5×10^5^) were treated for 24 h with 1, 10 and 50 µM α-cobratoxin from *N. kaouthia*.

### Animals

All the procedures involving animals were conducted as indicated in the Italian National Guidelines (D.L. No. 116 G.U., suppl. 40, 18.2.1992, circolare No. 8, G.U. July 1994) and in the appropriate European Directives (EEC Council Directive 86/609, 1.12.1987), adhering to the Guide for the Care and Use of Laboratory Animals (United States National Research Council, 1996) and according to an approved protocol reviewed by the Institutional Animal Care and Use Committee (Genova, 15 November 2004, reference N.149). All the *in vivo* experiments reported here were done on the basis of authorization no. 254 of the Animal Ethics Committee of the IST of Genoa (Italy).

CD1 female mice (Harlan Nossan, Milano, Italy), 6–7 weeks old, were used for toxicity studies. Mice were allowed a 7-day rest period before experiments. All mice were housed at 7–9 per cage, maintained at 22°C with a 12 h light/dark cycle and fed with a standard diet and water *ad libitum*.

Non-Obese Diabetic/Severe Combined Immunodeficient (*NOD/SCID*) mice (8 week-old) were used for toxin antitumor activity studies. They were born and housed in specific filter-capped cages, kept in pathogen-free conditions and maintained in the facilities within the animal resources centre at the IST of Genoa in accordance with the recommendations, regulations and standards approved by the Federation of European Laboratory Animal Science Association (FELASA).

### *In vivo* toxicity studies and identification of the Maximum tolerated dose (MTD)

Toxicity of α-neurotoxins was studied in CD1 mice. Animals were given intravenous injection (i.v.) of single toxin dose (range 0.1–5,0.mg/kg and 0.1–0.5 mg/kg for cobrotoxin and cobratoxin, respectively) and checked for acute toxicity. Fifteen minutes after dosing the mice were allowed free access to food and water. The behavior and number of survivors were checked daily over an observation period of 14 days. Body weight was recorded every 2 days and was used as an index of toxicity. Animals were sacrificed by CO_2_ at the end of the observation period and immediate autopsy was carried out.

The lethal dose (LD: the dose of a drug or treatment that will cause death) and maximum tolerated dose (MTD: the highest dose of a drug or treatment that does not cause unacceptable side effects) were identified.

### *In Vivo* orthotopic grafting of A549-luc human lung cancer cells and bioluminescence imaging (BLI) detection

*NOD/SCID* mice were xenografted with 0.5×10^6^ human A549-luc cells/20 µl PBS into the right lung [20]. Treatment started 7 days after surgery to allow tumor development and mimic clinical behavior. Experiments were carried out according to two different treatment protocols, as described in Results. Animals were sacrificed when they showed important symptoms of dyspnoea, fatigue, inability to reach food and water, emaciation and excessive decrease in weight loss. Each animal underwent autopsy to confirm the presence, characteristics and extent of tumor development.

Bioluminescence imaging (BLI) was carried out weekly by the *In Vivo* Imaging System (IVIS) technology (Caliper Life Sciences, Paris, France) which measures luciferase activity. Mice were injected i.p. with 200 µl Luciferin at 15 mg/ml (Promega Madison, Wisconsin, USA) 10 min before imaging and anesthetized by isoflurane. Thereafter, animals were put in the light-protected chamber of the IVIS imaging system, and photons emitted were measured over 3 min. Regions of interest (ROI) were drawn over the area of photon emission and quantified using the “Living Image” software.

## Supporting Information

Figure S1 **α7 NAchR expression in A549 and A549-luc cells treated with α–neurotoxins.** Cell lines were treated with 0.001, 0.003 and 0.009 µM **α**–cobratoxin and the expression of the **α**7 receptor was measured by qPCR utilizing as calibrator the RNA of untreated A549 or A549-luc and ß2 Microglobulin as reference gene. A: A549 **α**–cobratoxin, B: A549-luc **α**–cobratoxin; C: A549-luc **α**–cobrotoxin. N.D.: Not Determined.
(TIF)
