# Supplementary figures and images for: Inhibition of the Nicotinic Acetylcholine Receptors by Cobra Venom α-Neurotoxins: Is There a Perspective in Lung Cancer Treatment?
Source: PLoS One. 2011 Jun 13;6(6):e20695. doi: 10.1371/journal.pone.0020695 (PMC3113800; doi:10.1371/journal.pone.0020695)

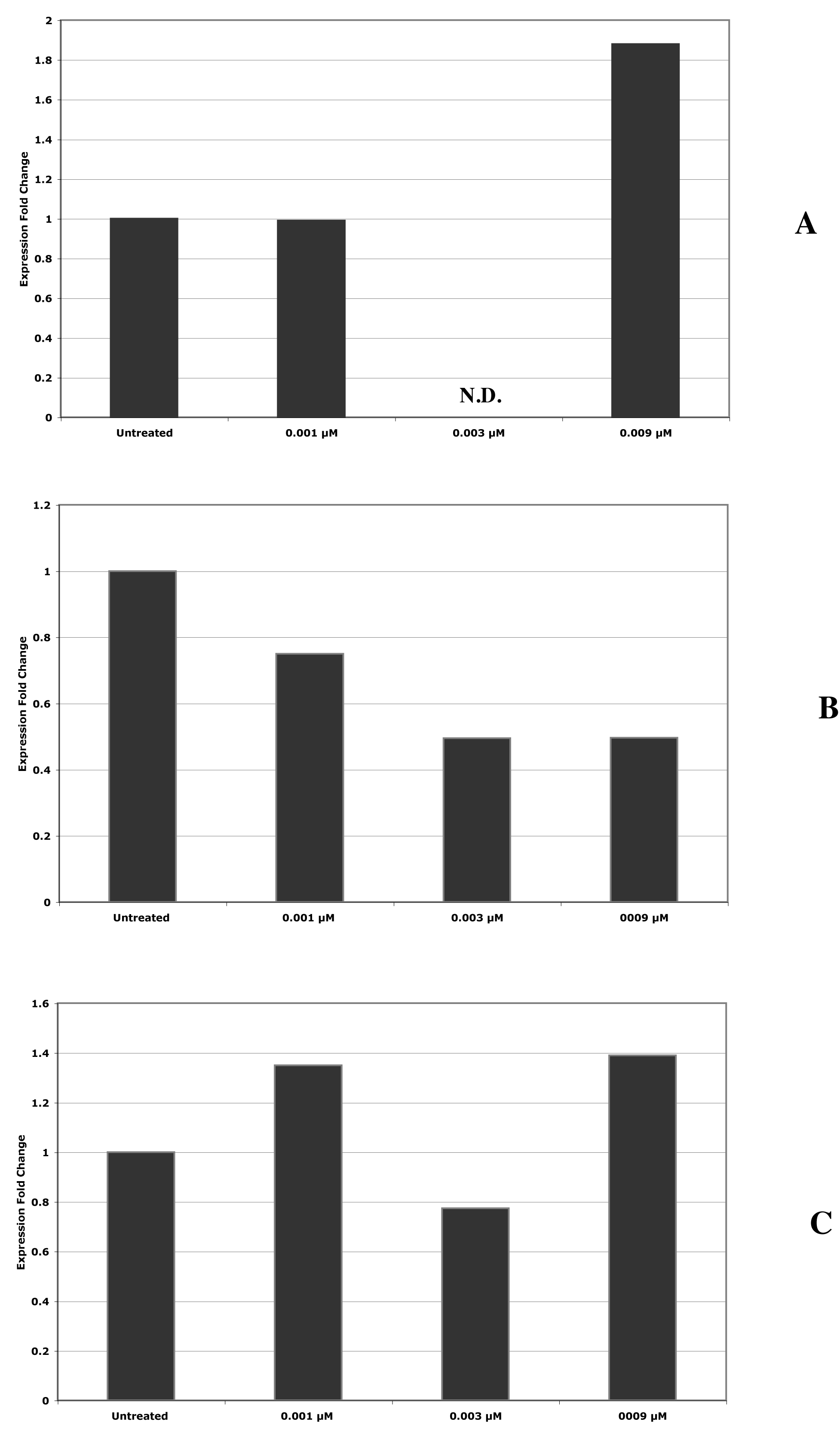

Supplement: Figure S1 — α7 NAchR expression in A549 and A549-luc cells treated with α–neurotoxins. Cell lines were treated with 0.001, 0.003 and 0.009 µM α–cobratoxin and the expression of the α7 receptor was measured by qPCR utilizing as calibrator the RNA of untreated A549 or A549-luc and ß2 Microglobulin as reference gene. A: A549 α–cobratoxin, B: A549-luc α–cobratoxin; C: A549-luc α–cobrotoxin. N.D.: Not Determined. (TIF) [file pone.0020695.s001.tif]
